# Supplementary material for: The negative cofactor 2 complex is a key regulator of drug resistance in Aspergillus fumigatus
Source: Nat Commun. 2020 Jan 22;11:427. doi: 10.1038/s41467-019-14191-1 (PMC7194077; doi:10.1038/s41467-019-14191-1)
Supplement: Supplementary file 1 — Supplementary Information [file 41467_2019_14191_MOESM1_ESM.pdf]

Supplementary Information

The Negative Cofactor 2 complex is a key regulator of drug resistance in *Aspergillus fumigatus.*

Authors:

Takanori Furukawa†[1,2], Norman van Rhijn†[1,2], Marcin Fraczek[1], Fabio Gsaller[1], Emma Davies[1], Paul Carr[1], Sara Gago[1,2], Rachael Fortune-Grant[1,2], Sayema Rahman[1,2], Jane Mabey Glisenan[1], Emma Houlder[2], Caitlin H. Kowalski[3], Shriya Raj[4], Sanjoy Paul[5], Peter Cook[2], Josie E. Parker[6], Steve Kelly[6], Robert A. Cramer[3], Jean-Paul Latge[4], Scott Moye-Rowley[5], Elaine Bignell[1,2], Paul Bowyer[1,2*], Michael J Bromley[1,2*]

Affiliations:

[1] Manchester Fungal Infection Group, Division of Infection, Immunity and Respiratory Medicine, Faculty of Biology, Medicine and Health, University of Manchester, CTF Building, 46 Grafton Street, Manchester, M13 9NT, UK.

[2] Lydia Becker Institute of Immunology and Inflammation, Manchester Collaborative Centre for Inflammation Research, Division of Infection, Immunity and Respiratory Medicine, Faculty of Biology, Medicine and Health, University of Manchester, Manchester Academic Health Science Centre, Manchester, UK.

[3] Department of Microbiology and Immunology, Geisel School of Medicine at Dartmouth, Hanover, NH, 03766, USA.

[4] Unité des Aspergillus , Institut Pasteur , 25 rue du Docteur Roux , 75724 Paris Cedex 15 , France.

[5] Department of Molecular Physiology and Biophysics, Carver College of Medicine, University of Iowa, Iowa City, IA, 52242, USA.

[6] Institute of Life Science, Swansea University Medical School, Swansea University, Swansea, Wales, SA2 8PP, UK.

[6] Department of Microbiology and Immunology, Geisel School of Medicine at Dartmouth, Hanover, NH, 03766, USA.

†These authors contributed equally.

* These authors jointly supervised this work.

To whom correspondence should be addressed:

Michael J Bromley*, Email: Mike.Bromley@manchester.ac.uk

Paul Bowyer*, Email: Paul.Bowyer@manchester.ac.uk

Supplementary Table 1. Identification of the interacting proteins of NctA and NctB using co-immunoprecipitation followed by liquid chromatography-spectrometory (LC-MS).

Protein identified	Accession Number	Molecular Weight	Number of peptides identified			Gene ID
			Control (native NctA/NctB)	NctA-S-tag	NctB-S-tag	
TBP associated factor (Mot1), putative	B0XPE7_ASPFC	210 kDa	0	19	3	AFUB_006220
CBF/NF-Y family transcription factor, putative (NctA)	B0XTT5_ASPFC	27 kDa	0	11	3	AFUB_029870
40S ribosomal protein S24	B0YC29_ASPFC	15 kDa	0	7	5	AFUB_088700
60S ribosomal protein L27a, putative	B0XZ73_ASPFC	17 kDa	0	6	2	AFUB_043390
60S ribosomal protein L20	B0XNN1_ASPFC	20 kDa	0	5	5	AFUB_004870
Ribosomal protein L26	B0Y8G7_ASPFC	18 kDa	0	4		AFUB_077280
Hsp70 chaperone Hsp88	B0XR33_ASPFC	80 kDa	0	4		AFUB_012080
Glyceraldehyde-3-phosphate dehydrogenase	B0Y207_ASPFC	36 kDa	0	4	4	AFUB_050490
Ribosomal protein	B0XQU0_ASPFC	24 kDa	0	3	4	AFUB_011140
60S ribosomal protein L5	B0XR75_ASPFC	35 kDa	0	3	3	AFUB_012370
Casein kinase I, putative	B0XTZ5_ASPFC	46 kDa	0	3		AFUB_019630
Ctr copper transporter family protein	B0XUP5_ASPFC	28 kDa	0	3		AFUB_020800
Transcriptional corepressor Cyc8, putative	B0XSL1_ASPFC	95 kDa	0	3		AFUB_027580
60S Ribosomal protein L37	B0XYW1_ASPFC	11 kDa	0	3	2	AFUB_042280
60S ribosomal protein L8	B0Y3E2_ASPFC	27 kDa	0	3	3	AFUB_053890
Alanine--tRNA ligase	B0YA78_ASPFC	107 kDa	0	3		AFUB_083700
CBF/NF-Y family transcription factor, (NctB)	B0Y0F3_ASPFC	16 kDa	0	3	4	AFUB_045980
ATP citrate lyase, subunit 1, putative	B0Y8A8_ASPFC	72 kDa	0	2	3	AFUB_076690
UTP-glucose-1-phosphate uridylyltransferase Ugp1, putative	B0YBZ9_ASPFC	58 kDa	0	2	7	AFUB_088400
Ribosome associated DnaJ chaperone Zuotin, putative	B0YEJ2_ASPFC	51 kDa	0	2	3	AFUB_099370
Eukaryotic translation initiation factor 3 subunit J	EIF3J_ASPFC	30 kDa	0	2	4	AFUB_072690
Nitrilase	B0XM85_ASPFC	45 kDa	0		6	AFUB_001300
Mitochondrial aconitate hydratase, putative	B0XM04_ASPFC	85 kDa	0		3	AFUB_001810
14-3-3 family protein ArtA, putative	B0XUD6_ASPFC	29 kDa	0		4	AFUB_020360
Nucleolar protein nop5	B0XWG2_ASPFC	65 kDa	0		3	AFUB_035810
ATP citrate lyase subunit (Acl), putative	B0Y8A9_ASPFC	53 kDa	0		3	AFUB_076700
Putative uncharacterized protein	B0YAT7_ASPFC	105 kDa	0		3	AFUB_085790

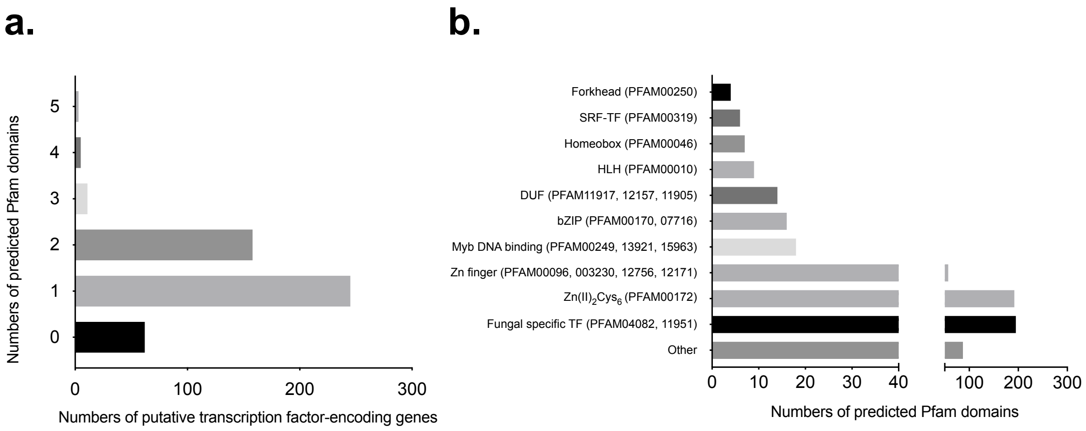

Supplementary Figure 1: Overview of the identified 495 putative transcription factors in the *A. fumigatus* A1163 genome. (a) Domain compositions of the putative transcription factors. The number of predicted Pfam domains in a single transcription factor is shown. **(b)** Distribution of *A. fumigatus* transcription factors among the fungal class of Pfam regulator families. The Pfam IDs used for the domain search analysis are shown in the graph. Source data are provided as a Source Data file.

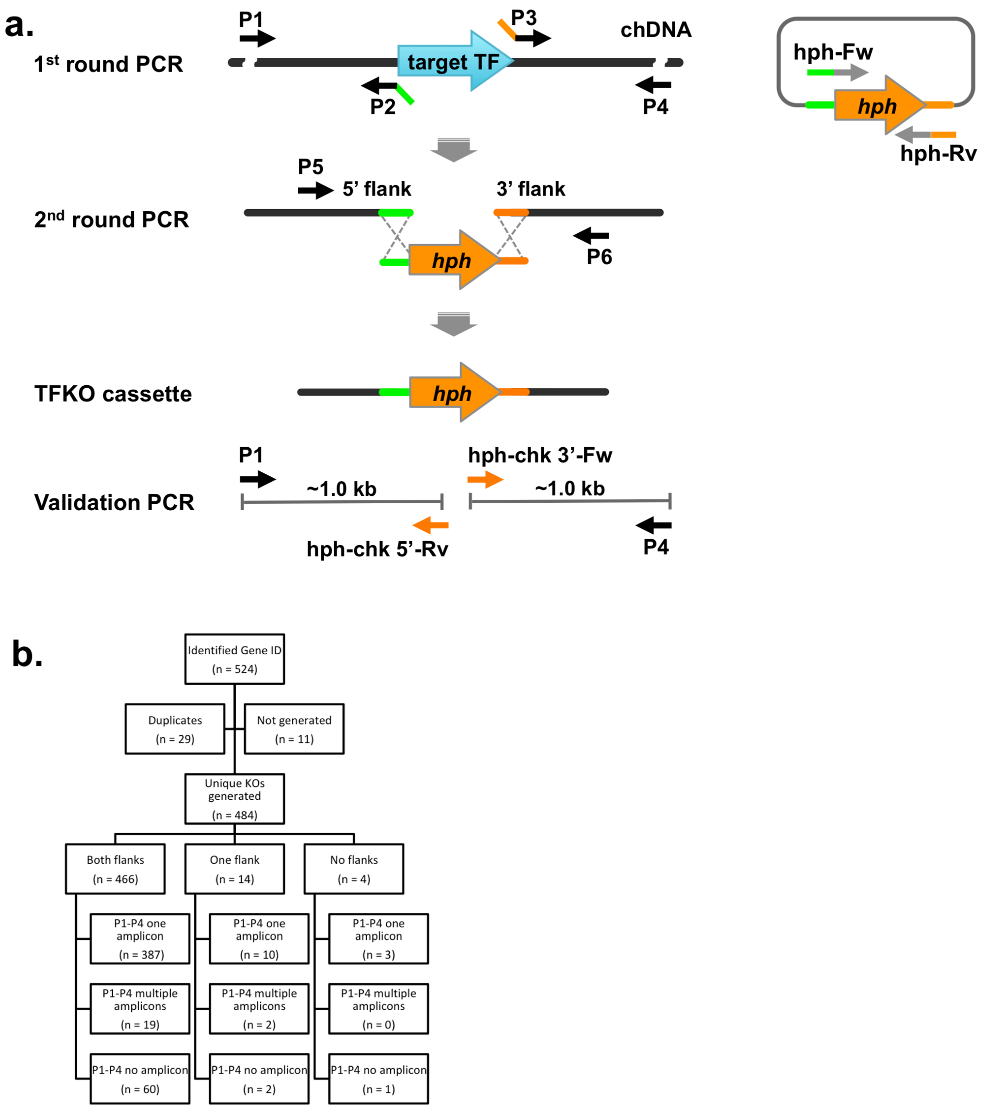

Supplementary Figure 2: Generation of *A. fumigatus* transcription factor knockout mutants. (a) Schematic diagram of the construction of a transcription factor knockout (TFKO) cassette by fusion PCR. The flanking regions of the target gene are amplified independently from the chromosomal DNA using the primer pairs P1/P2 and P3/P4, respectively. Primers P2 and P3 are designed to include a 20-bp of 5'-tail (shown in green and orange) homologous to the ends of the hygromycin resistance (*hph*) marker cassette. Each pre-amplified fragment is fused together by fusion PCR using nested primers P5 and P6 to give a TFKO cassette. Homologous integration of the TFKO cassette was verified by PCR using the primer pairs P1/hph-chk-57-Rv (for 5'-junction), hph-chk 3'-Fw/P4 (for 3'-junction), and P1/P4 (for entire TFKO cassette). **(b)** Flowchart describing the construction of the *A. fumigatus* TFKO library and its quality control process.

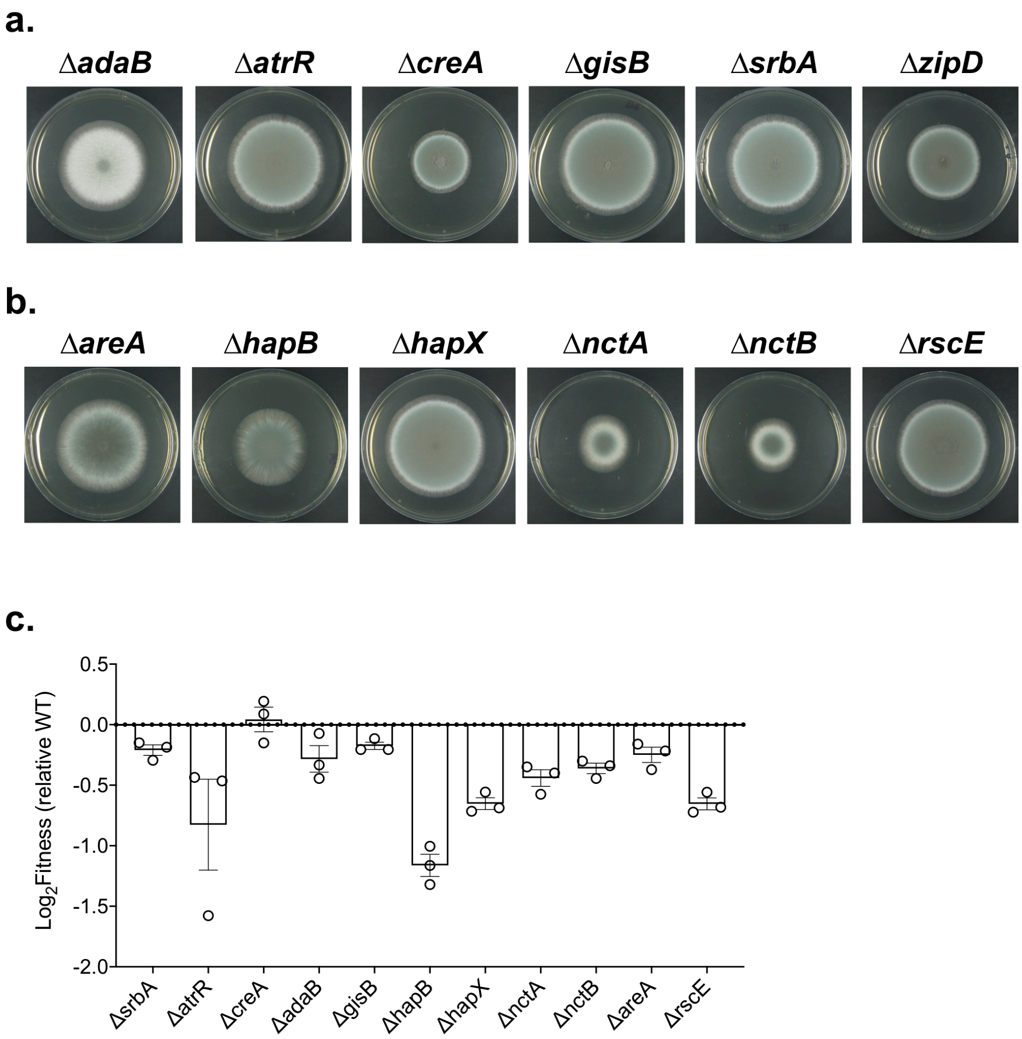

Supplementary Figure 3: Growth phenotypes of the identified key transcription factor null mutants associated with itraconazole sensitivity and resistance. (a and c) Colonial growth phenotypes of **(a)** the itraconazole sensitive and (**b**) the itraconazole resistance transcription factor null mutants on a solid Aspergillus complete medium (ACM) after 72 h at 37 ˚C. **(c)** Relative growth fitness of the identified transcription factor null mutants to the wiled-type stain measured in RPMI-1640 liquid medium. Data are represented as mean ± SEM. Source data are provided as a Source Data file.

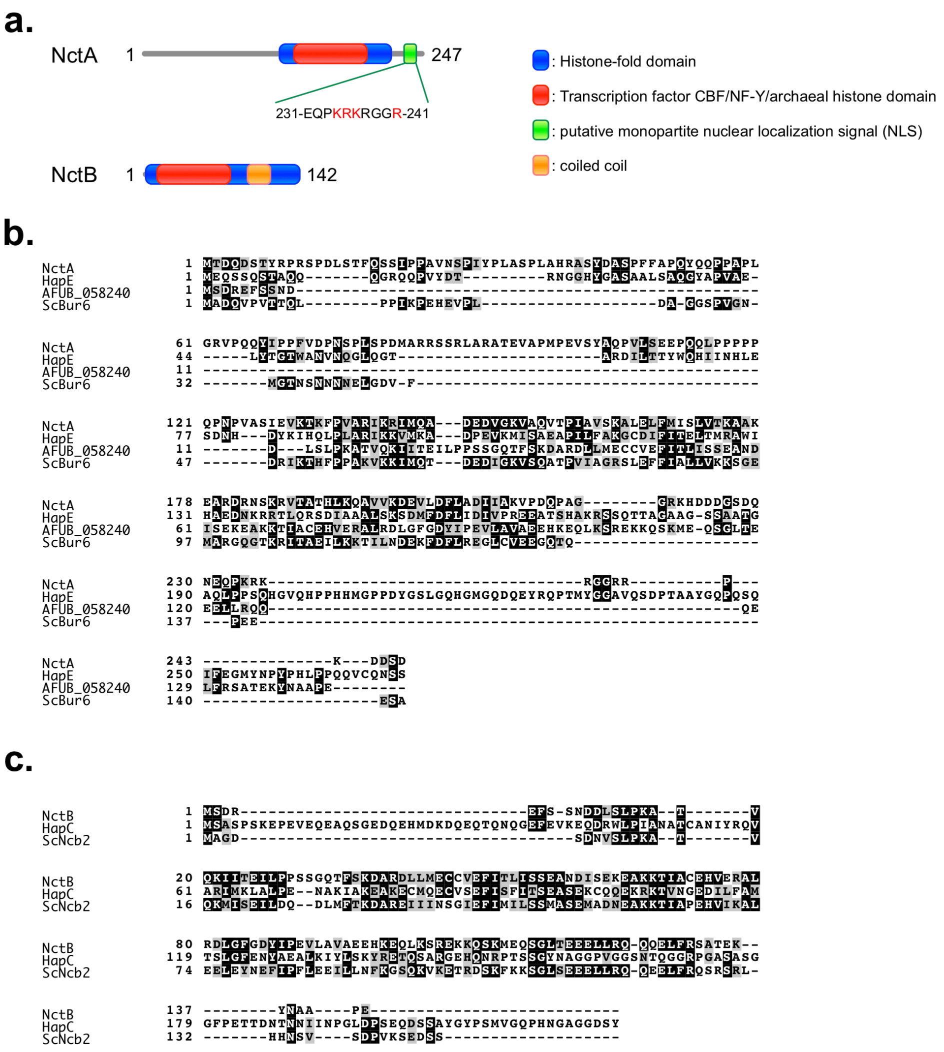

Supplementary Figure 4: Domain structure and sequence alignment of NctA and NctB. **(a)** Schematic representation of the domain structure of *A. fumigatus* NctA and NctB. The histone-fold domain (IPR009072) predicted by InterPro Scan is shown in blue. The transcription factor CBF/NF-Y/archaeal histone domain (PF00808) predicted by PfamScan is shown in red. The position of the putative monopartite nuclear localization signal (NLS) identified by NLS-mapper within the C-terminal region of NctA is shown in green with amino acid sequence, and the predicted coil region found within the histone-hold domain of NctB is depicted in orange, respectively. **(b)** Multiple protein sequence alignment of *A. fumigatus* NctA, its paralogues (HapE and AFUB_058240), and the orthologue Bur6 in *Saccharomyces cerevisiae*. **(c)** Multiple protein sequence alignment of *A. fumigatus* NctB, its paralogue (HapC), and the orthologue Ncb2 in *S. cerevisiae*.

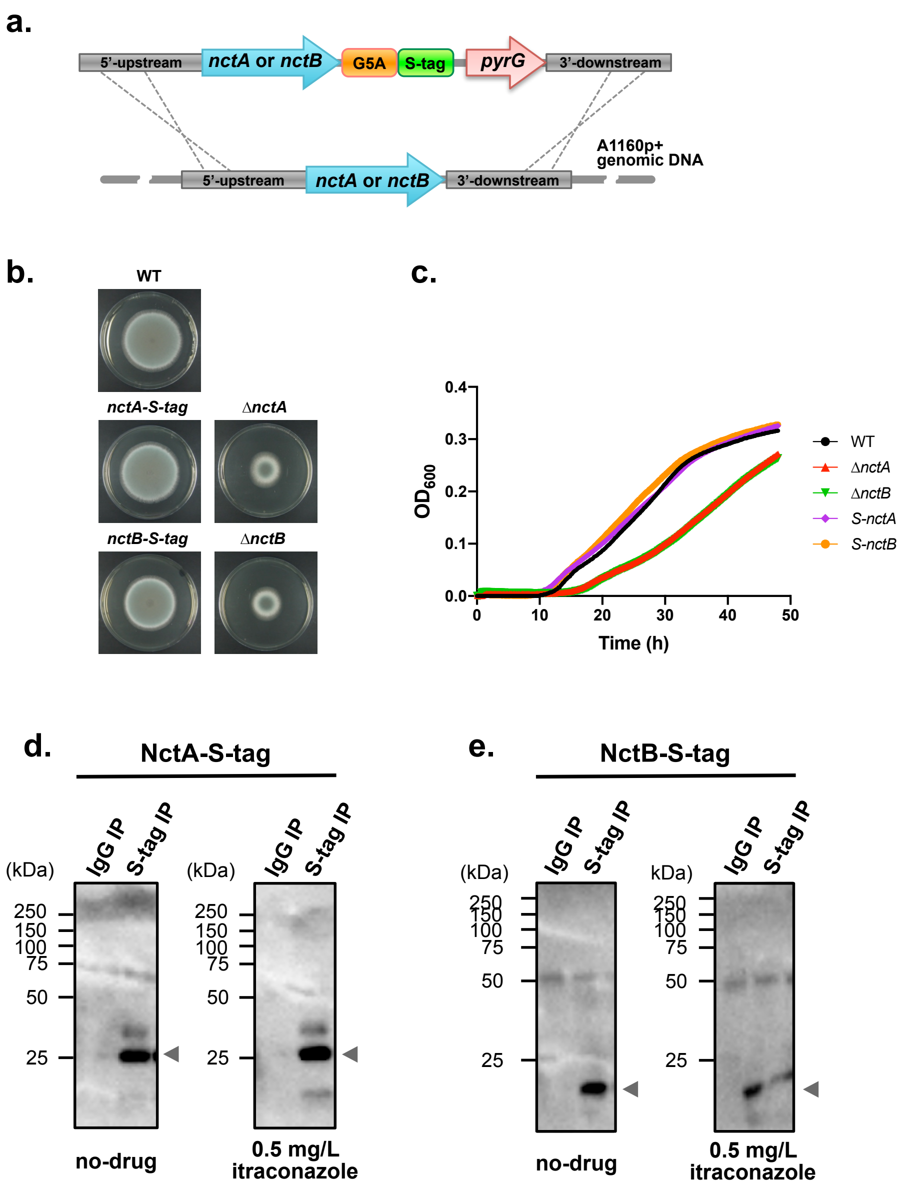

Supplementary Figure 5: Validation of chromatin immunoprecipitation (ChIP) conditions for the C-terminally S-tagged derivative of NctA (NctA-S-tag). (a) Schematic representation of the construction of the S-tagged NctA and NctB expressing strains. **(b and c)** Growth phenotypes of the S-tagged NctA and NctB expressing strains **(b)** on a solid ACM after 72 h at 37 ˚C and **(c)** in RPMI-1640 liquid medium. The mutant strains were cultivated in a microtiter plate containing 200 μL of a fungal culture medium and incubated at 37 °C. Optical density at 600 nm was measured at every 10 minute for a total period of 48 hours. The growth analysis was performed on three separate occasions. **(d and e)** Immunoprecipitation followed by Western blotting (IP-WB) analysis of **(d)** the NctA-S-tag and **(e)** the NctB-S-tag expressing strain. Cell-free extract of the NctA-S-tag expressing mutant prepared from no-drug and 0.5 mg/L itraconazole conditions were immunoprecipitated with a S-tag specific polyclonal antibody (S-tag IP) or a control rabbit IgG (IgG IP), and then subjected to WB. Specific signals derived from NctA-S-tag protein is indicated by triangles. The estimated molecular weight of NctA-S-tag is 30.2 kDa and NctB-S-tag is 19.1 kDa. Source data are provided as a Source Data file.

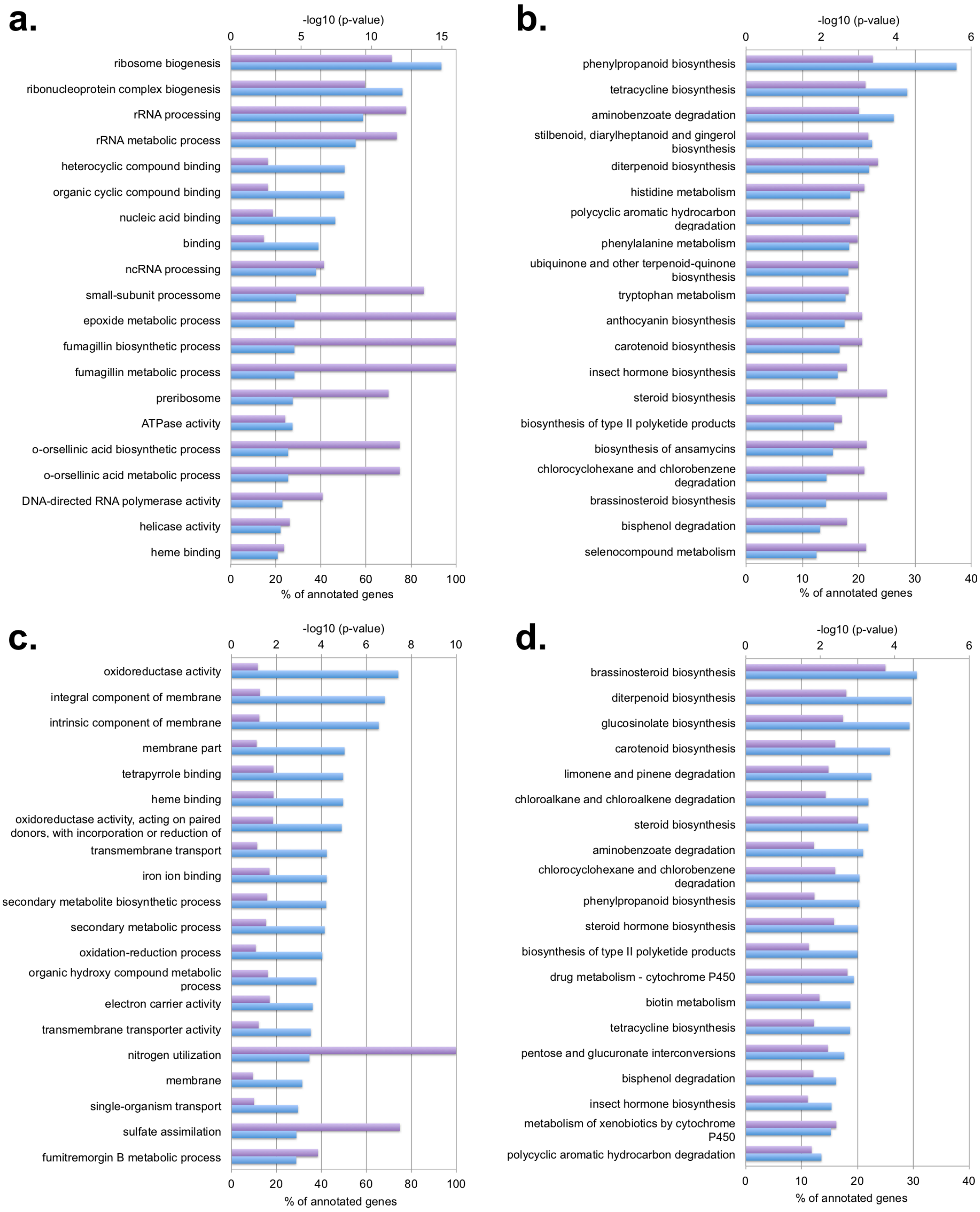

Supplementary Figure 6: NCT complex is a global regulator of diverse biological processes including secondary metabolism and steroid biosynthesis. Gene Ontology (GO) term **(a and c)** and KEGG pathway **(b and d)** enrichment analysis of *A. fumigatus* genes that are differentially expressed in the *nctA* null mutant compared to the wild-type. The top 20 significantly over-represented GO terms and KEGG pathways in **(a and b)** no-drug and **(c and d)** 0.5 mg/L itraconazole conditions are shown. The percentage of differentially expressed genes in each term (purple bars) and their statistical significance (–log10 *p*-values, blue bars) are plotted on the x-axis. Significance level of the enrichment was analyzed using the Benjamini-Hochberg adjustment method with a *p*-value cutoff <0.05. Source data are provided as a Source Data file.

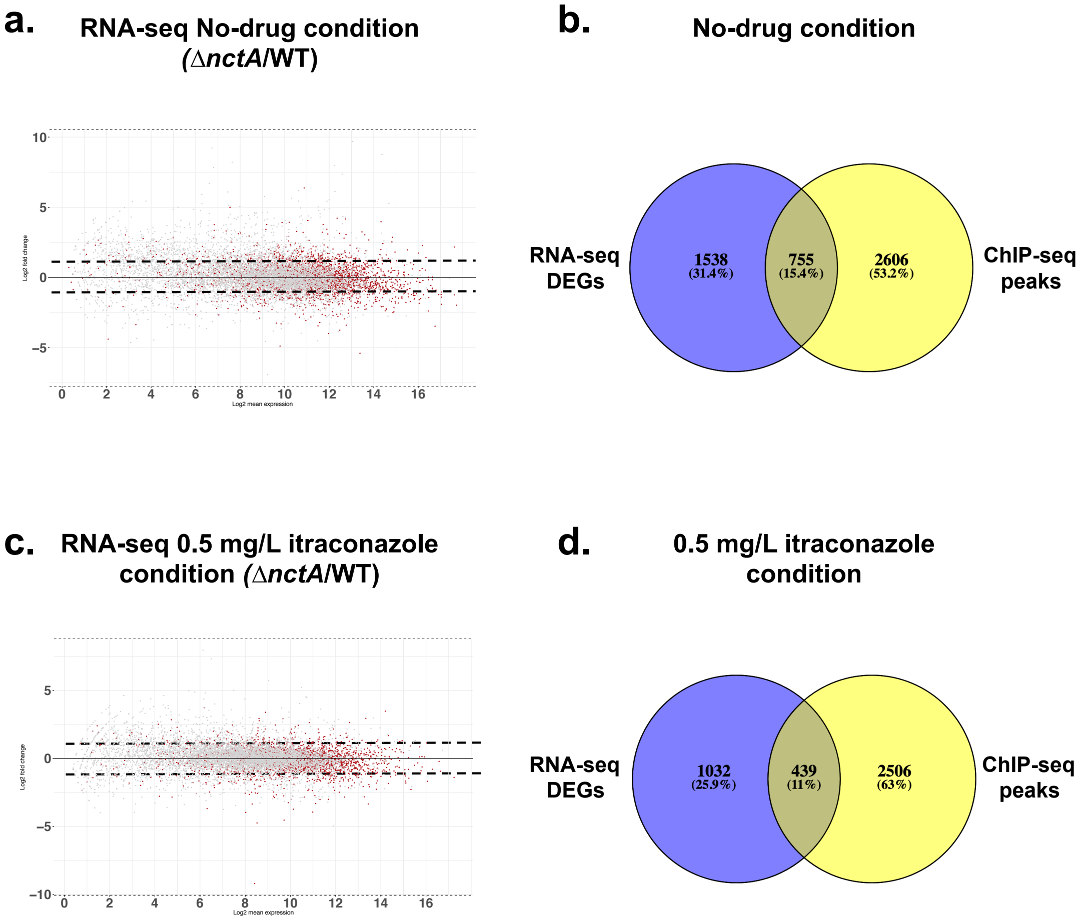

Supplementary Figure 7. Correlation between NctA occupancy and gene expression changes. **(a, c)** MA-plot showing correlation between NctA occupancy and gene expression changes in the *nctA* null mutant in **(a)** no-drug and **(c)** 0.5 mg/L itraconazole conditions. Genes with a significant NctA binding peak (p-value<0.01, fold enrichment >1.5) are indicated with red dots. **(b,d)** Venn diagram showing overlap of genes that are differentially expressed in the *nctA* null mutant, and genes with significant NctA ChIP-seq peaks in **(b)** no-drug and **(d)** 0.5 mg/L itraconazole conditions.

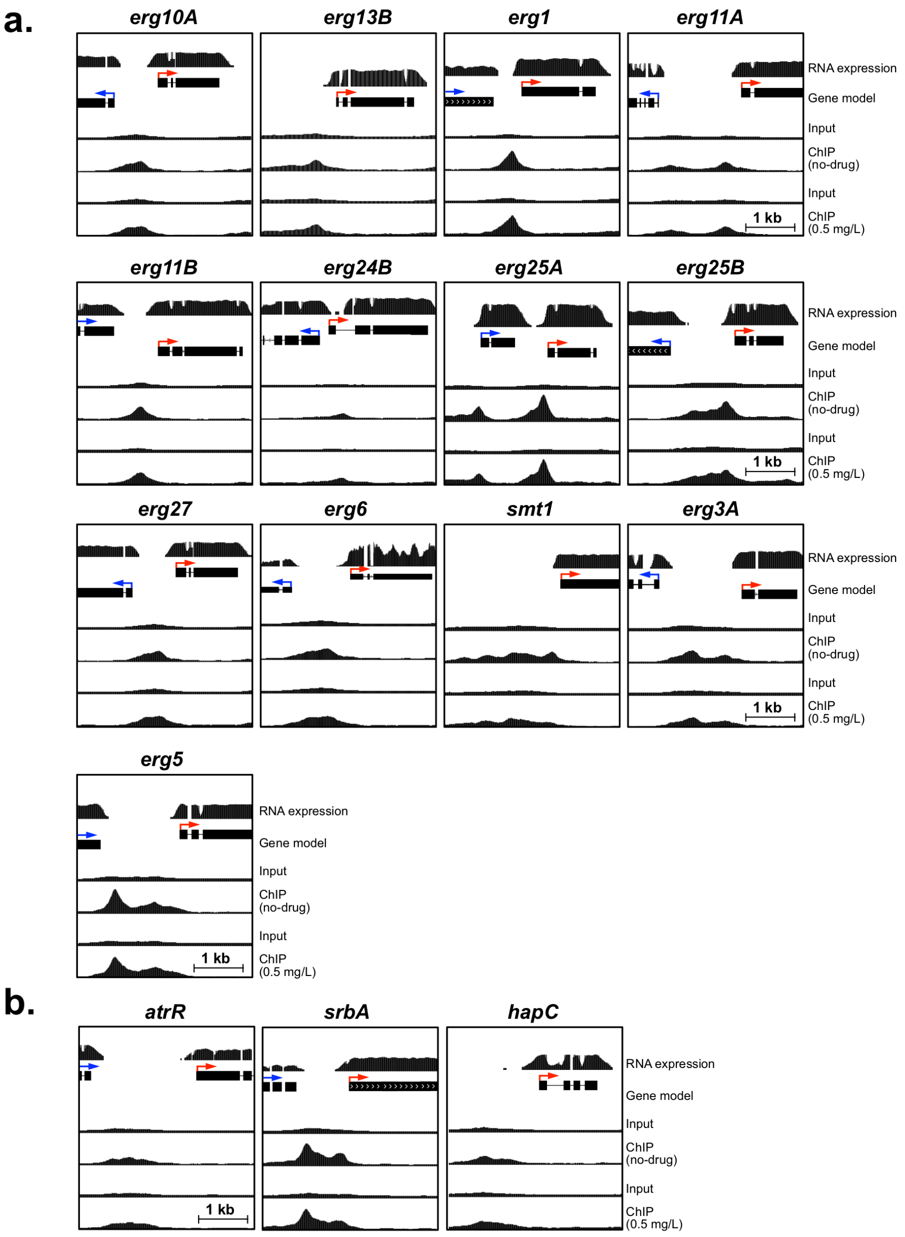

Supplementary Figure 8. Binding of NctA on the 5'-upstream region of the genes involved in ergosterol biosynthesis and their known transcriptional regulators. *In vivo* binding of NctA on the 5'-upstream region of **(a)** ergosterol biosynthetic genes, and **(b)** their known transcriptional regulators. Tracks for the NctA ChIP-seq (ChIP) and their input DNA control (Input) are visualized in the UCSC genome browser together with annotated gene models and their transcript, which are expressed in the no-drug conditions. Direction of the target gene and the 5'-proximal gene are shown in red arrows and blue arrows, respectively.

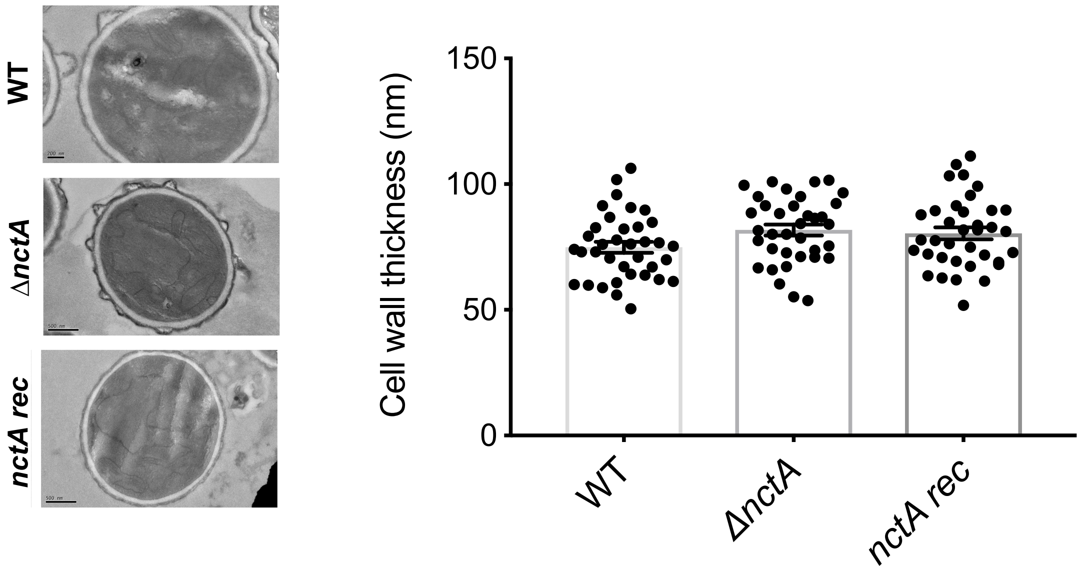

Supplementary Figure 9. Loss of NCT function doesn't affect cell wall thickness. Width of the cell wall layer of the conidia determined by TEM image analysis are shown. The data represents the mean of 36 conidia of each strain. Data are represented as mean ± SEM. Source data are provided as a Source Data file.

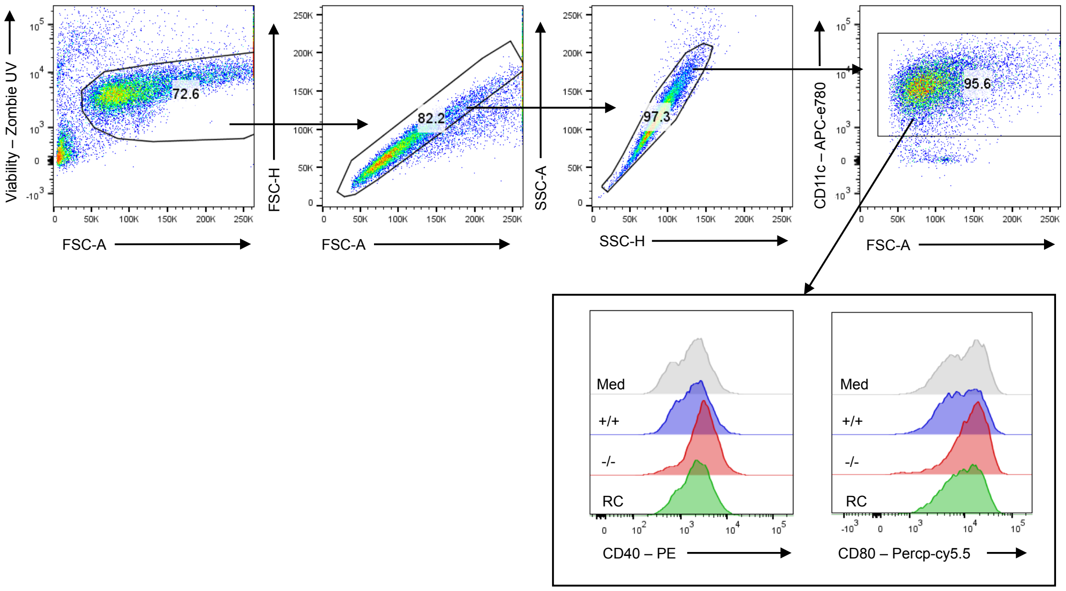

Supplementary Figure 10. Representative flow cytometry gating strategy for Figure 8, showing gating of dendritic cells to assay activation. Approximately 10,000 CD11c^{+} dendritic cells were assayed for each replicate.
