# Supplementary material for: The negative cofactor 2 complex is a key regulator of drug resistance in Aspergillus fumigatus
Source: Nat Commun. 2020 Jan 22;11:427. doi: 10.1038/s41467-019-14191-1 (PMC7194077; doi:10.1038/s41467-019-14191-1)
Supplement: Supplementary file 3 — Description of Additional Supplementary Files [file 41467_2019_14191_MOESM3_ESM.pdf]

Description of Additional Supplementary Files

File Name: Supplementary Data 1
Description: Transcription factors identified in the A. fumigatus genome, and the construction of the transcription factor knockout library. Tab1: List of the identified putative transcription factors and a summary of transcription factor knockout library. Tab2: Primers used in this study. Tab 3: Raw output of HMMR transcription factor domain search. Tab 4: Manual analysis of the putative transcription factor encoding genes without Pfam DNA binding domains.

File Name: Supplementary Data 2
Description: List of differentially expressed genes identified by RNA-seq in the nctA null and the nctB null mutants. Tab 1-4: Output of DESeq differential expression analysis. Tab 5: Dysregulated secondary metabolite gene clusters. Tab 6: Expression profiles of the genes involved in the ergosterol biosynthesis pathway. Tab 7: Expression profiles of the transcription factor genes associated with azole tolerance, mot1, and siderophore biosynthesis related genes.

File Name: Supplementary Data 3
Description: Genome-wide identification of NctA binding regions and the correlation between NctA occupancy and gene expression changes. Tab1: Summary of peak calling for the NctA ChIP-seq datasets. Tab2: Genome-wide binding profiles of NctA in nodrug conditions. Tab3: Genome-wide binding profiles of NctA in 0.5 mg/L itraconazole conditions.
